# CT-2A neurospheres-derived high-grade glioma in mice: a new model to address tumor stem cells and immunosuppression

**DOI:** 10.1242/bio.044552

**Published:** 2019-09-11

**Authors:** Matteo Riva, Roxanne Wouters, Akila Weerasekera, Sarah Belderbos, David Nittner, Dietmar R. Thal, Thaïs Baert, Roberto Giovannoni, Willy Gsell, Uwe Himmelreich, Marc Van Ranst, An Coosemans

**Affiliations:** 1 Department of Oncology, Laboratory of Tumor Immunology and Immunotherapy, KU Leuven, Leuven 3000, Belgium; 2 Department of Neurosurgery, Erasme Hospital, Bruxelles 1070, Belgium; 3 Biomedical MRI, Department of Imaging and Pathology and Molecular Small Animal Imaging Center (MoSAIC), KU Leuven, Leuven 3000, Belgium; 4 Center for the Biology of Disease, KU Leuven Center for Human Genetics - InfraMouse, VIB, University of Leuven, Leuven 3000, Belgium; 5 Laboratory of Neuropathology, Department of Imaging and Pathology, Leuven Brain Institute, KU Leuven, Leuven 3000, Belgium; 6 Department of Pathology, UZ-Leuven, Leuven 3000, Belgium; 7 Department of Gynecology and Gynecologic Oncology, Kliniken Essen Mitte (KEM), Essen 2910, Germany; 8 Department of Medicine and Surgery, University of Milano-Bicocca, Monza 20900, Italy; 9 Laboratory for Clinical and Epidemiological Virology, Rega Institute for Medical Research, KU Leuven, Leuven 3000, Belgium; 10 Department of Gynaecology and Obstetrics, Leuven Cancer Institute, UZ Leuven, Leuven 3000, Belgium

**Keywords:** High-grade glioma model, CT-2A, Glioma stem cells, Immunosuppression, Tregs

## Abstract

Recently, several promising treatments for high-grade gliomas (HGGs) failed to provide significant benefit when translated from the preclinical setting to patients. Improving the animal models is fundamental to overcoming this translational gap. To address this need, we developed and comprehensively characterized a new *in vivo* model based on the orthotopic implantation of CT-2A cells cultured in neurospheres (NS/CT-2A). Murine CT-2A methylcholanthrene-induced HGG cells (C57BL/6 background) were cultured in monolayers (ML) or NS and orthotopically inoculated in syngeneic animals. ML/CT-2A and NS/CT-2A tumors' characterization included the analysis of tumor growth, immune microenvironment, glioma stem cells (GSCs), vascularization and metabolites. The immuno-modulating properties of NS/CT-2A and ML/CT-2A cells on splenocytes were tested *in vitro*. Mice harboring NS/CT-2A tumors had a shorter survival than those harboring ML/CT-2A tumors (*P*=0.0033). Compared to standard ML/CT-2A tumors, NS/CT-2A tumors showed more abundant GSCs (*P*=0.0002 and 0.0770 for Nestin and CD133, respectively) and regulatory T cells (Tregs, *P*=0.0074), and a strong tendency towards an increased vascularization (*P*=0.0503). There were no significant differences in metabolites' composition between NS/ and ML/CT-2A tumors. *In vitro*, NS were able to drive splenocytes towards a more immunosuppressive status by reducing CD8^+^ T cells (*P*=0.0354) and by promoting Tregs (*P*=0.0082), macrophages (MF, *P*=0.0019) and their M2 subset (*P*=0.0536). Compared to standard ML/CT-2A tumors, NS/CT-2A tumors show a more aggressive phenotype with increased immunosuppression and GSCs proliferation. Because of these specific features, the NS/CT-2A model represents a clinically relevant platform in the search for new HGG treatments aimed at reducing immunosuppression and eliminating GSCs.

## Abstract (precis)

**Summary:** The NS/CT-2A tumor model represents a valuable research platform for the study of innovative treatments aimed at eliminating GSCs and reversing the tumor-induced immunosuppression in HGGs.

## INTRODUCTION

High-grade gliomas (HGGs) are aggressive brain tumors. With current standard therapies (surgery and chemo-radiation), the median survival of glioblastoma (GBM, the most malignant subtype of HGG) is only 15 months, relapse is almost universal and the large majority of patients ultimately die of the disease (Ostrom et al., 2014). During the last few decades, a vast amount of research has been carried out and the knowledge of HGG biology has tremendously improved. This has led to the development of several innovative treatments, such as immunotherapy or oncolytic virotherapy; nevertheless, despite very promising preclinical results, all these therapies failed to provide significant survival benefits when administered to patients (Dunn-Pirio and Vlahovic, 2017; Filley et al., 2017; Luksik et al., 2017). The inability of animal models to accurately mimic the clinical scenario probably played a relevant role in weakening the validity of preclinical studies and drastically reducing their translational impact (Guishard et al., 2017). Therefore, building new and more accurate *in vivo* models is essential to develop more effective treatments against HGGs.

The murine CT-2A cell line was established in C57BL/6J mice by Seyfried and colleagues in 1992 (Seyfried et al., 1992). Monolayer(ML)-cultured CT-2A (ML/CT-2A) cells implanted in immune-competent C57BL/6J mice give rise to orthotopic HGGs (Martinez-Murillo and Martinez, 2007). More recently, Binello and colleagues demonstrated that culturing CT-2A cells in neurospheres (NS/CT-2A) induces an increase of the glioma stem cell (GSC) population compared to the standard ML conditions (Binello et al., 2012). Similar to ML/CT-2A, NS/CT-2A cells were also able to generate brain tumors *in vivo*; however, such tumors have not been characterized. In particular, it still remains to be understood whether the differences between ML/ and NS/CT-2A cells are limited to the *in vitro* setting or if they could also have an impact on the *in vivo* development of brain tumors. In this study, we aimed at answering this question by investigating the differences in the biological behavior of NS/ and ML/CT-2A tumors.

## RESULTS

### CT-2A NS induce faster tumor growth

**Fig. 1.**
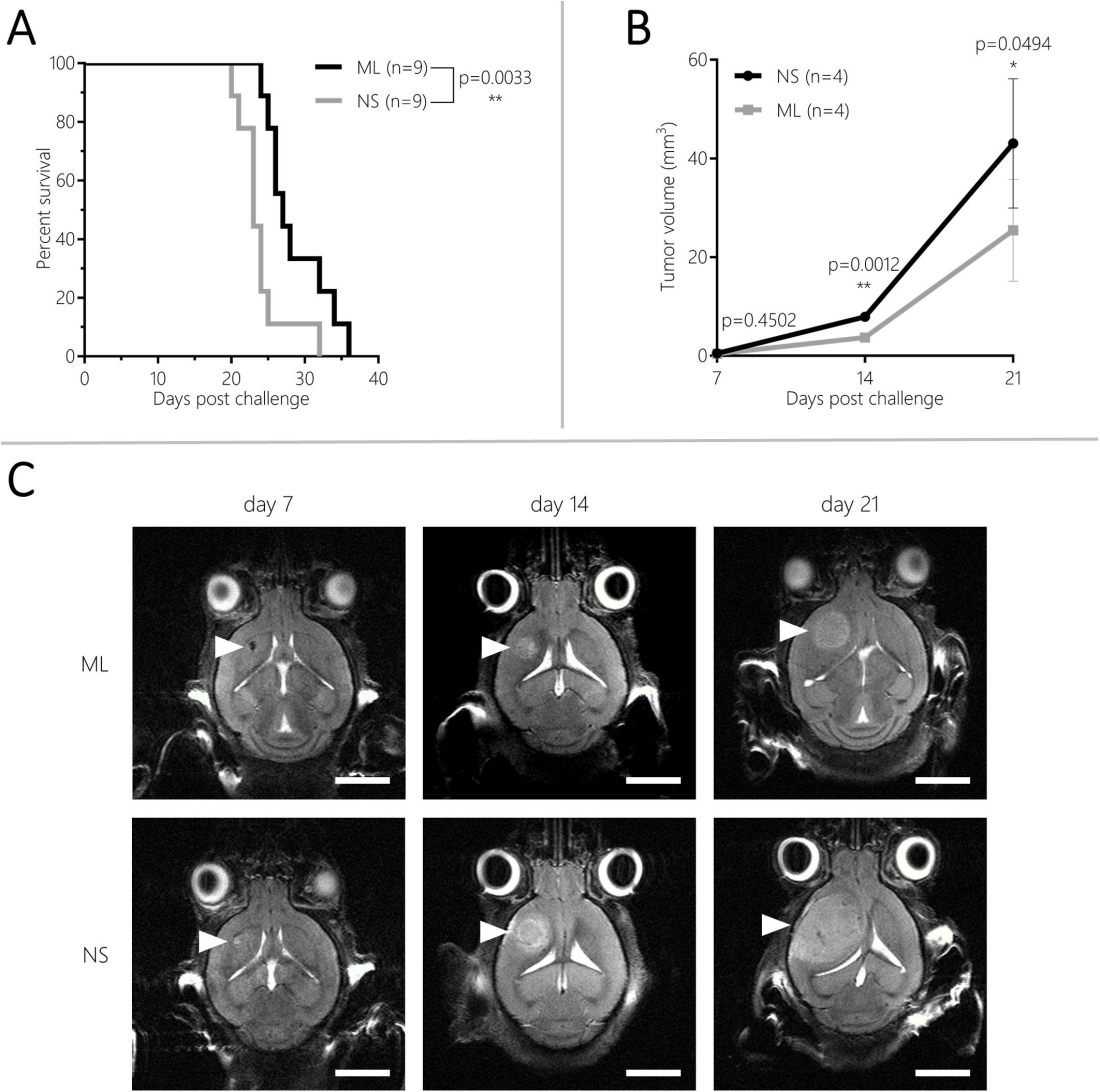
Eighteen mice (nine per group) were inoculated with 5×10^3^ NS/ or ML/CT-2A cells and were followed up clinically for survival analysis. Mice inoculated with NS/CT-2A cells survived significantly shorter than mice inoculated with ML/CT-2A (A). Eight random mice (four per group) were imaged with T2-weighted MRI on day 7, 14 and 21 after tumor cells inoculation for the analysis of tumor growth (B). On day 14 and 21 after tumor cells inoculation, NS/CT-2A tumors were significantly larger than ML/CT-2A tumors. Representative T2-weighted MRI images of NS and ML/CT-2A tumor are available in C (scale bars: 0.5 cm). NS, NS/CT-2A tumors; ML, ML/CT-2A tumors.

Survival and tumor volume of mice bearing NS/CT-2A and ML/CT-2A tumors were compared in order to analyze whether NS culture was able to change tumor behavior *in vivo*. All mice inoculated with 5×10^3^ NS/ or ML/CT-2A cells developed brain tumors. Mice harboring NS/CT-2A tumors had a significantly shorter survival compared to those harboring ML/CT-2A tumors (*n*=9 per group, median survival 23 versus 27 days, respectively, *P*=0.0033; Fig. 1A). Of note, 15 mice were euthanized upon achievement of the weight endpoint whereas three mice were euthanized upon development of grade 3 symptoms. Four mice per group were used for the longitudinal analysis of tumor volume. Seven days after tumor cells inoculation, a very small mass was already visible but no significant difference was found between NS/CT-2A and ML/CT-2A tumors (*P*=0.45). Fourteen and 21 days after tumor cells inoculation, NS/CT-2A tumors were significantly larger than their ML counterpart (mean volume 7.9±0.8 versus 3.2±1.1 mm^3^, respectively, *P*=0.0012, on day 14 and 43.1±11.3 versus 20.8±10.9 mm^3^, respectively, *P*=0.0494, on day 21; Fig. 1B–C). Individual tumor growth curves are available in the supplementary material (Fig. S1). The analysis of Hematoxylin and Eosin (H&E) staining of NS/CT-2A tumors revealed the presence of invasive, malignant and highly proliferative (21 mitotic figures counted in ten HPF) brain neoplasms. Furthermore, the diffuse expression of glial fibrillary acidic protein (GFAP) indicated the glial origin of such tumors (Fig. S2). Although some typical hallmarks of GBMs were lacking, such as microvascular proliferation and pseudopalisading necrosis, the high mitotic activity and the weak expression of GFAP indicate that this tumor biologically equals a high-grade astrocytoma, in the light of the high mitotic activity probably in transition into a GBM.

### CT-2A NS promote GSCs and tumor vasculature

**Fig. 2.**
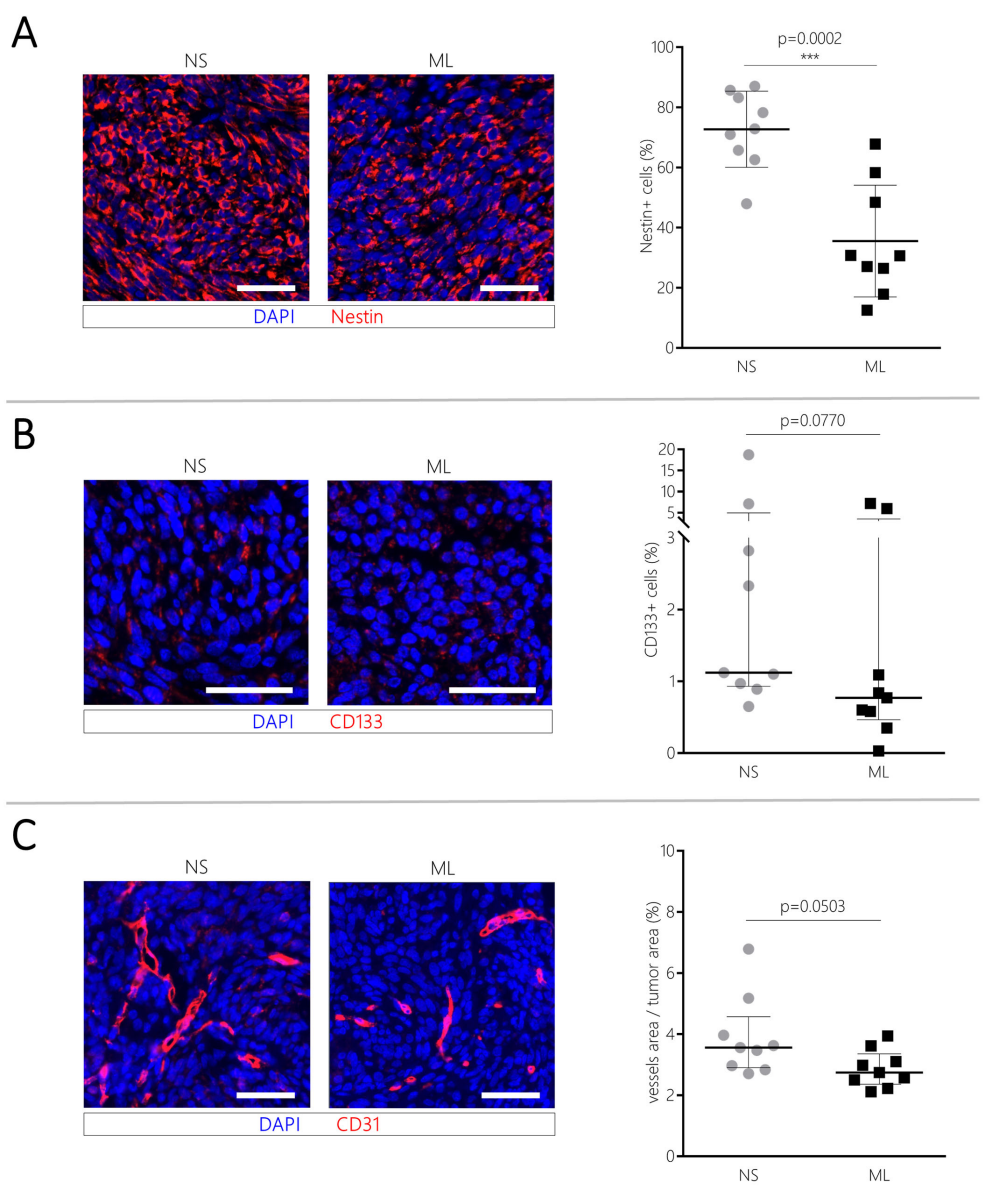
**GSCs and tumor vasculature.** Six mice (three per group) were inoculated with 5×10^3^NS/ or ML/CT-2A cells. 19 days after tumor cells inoculation, mice were euthanized, the whole brains were collected and stained for IHC. From each brain, three tumor sections were stained and analyzed. NS/CT-2A tumors showed a significantly higher expression of the GSC markers Nestin (A), a trend towards an higher expression of the GSC marker CD133 (B) and a strong trend towards a significant higher expression of the endothelial marker CD31 (C) compared to ML/CT-2A tumors. Left panels: representative images of fluorescence immunohistochemistry (scale bars: 50 μm); right panels: quantification of markers' expression with the software QuPath. NS, NS/CT-2A tumors; ML, ML/CT-2A tumors; CD, cluster of differentiation.

We evaluated the presence of GSCs within the tumor by staining the brains for Nestin and CD133. Nine brain sections from three different tumor-bearing mice were analyzed for both NS/CT-2A and ML/CT-2A tumors. Compared to their ML counterpart, NS/CT-2A tumors showed a significantly larger amount of Nestin-positive cells (35.5±6.2% versus 72.7±4.2%, respectively, *P*=0.0002; Fig. 2A). The same tendency was true for Cluster of Differentiation (CD) 133, although the total amount of CD133-positive cells was much lower compared to Nestin [1.12% (0.95–4.97) versus 0.77% (0.465–3.525), respectively, *P*=0.070; Fig. 2B]. CD31 staining was used to evaluate blood vessels within the tumors. A strong trend towards an increased ratio between vascular and tumor area [3.6% (2.9–4.6) versus 2.7% (2.4–3.4), respectively, *P*=0.0503; Fig. 2C] was seen in NS/CT-2A tumors compared to ML/CT-2A tumors.

### CT-2A NS modulate the tumor microenvironment towards increased suppression of the adaptive immune system

**Fig. 3.**
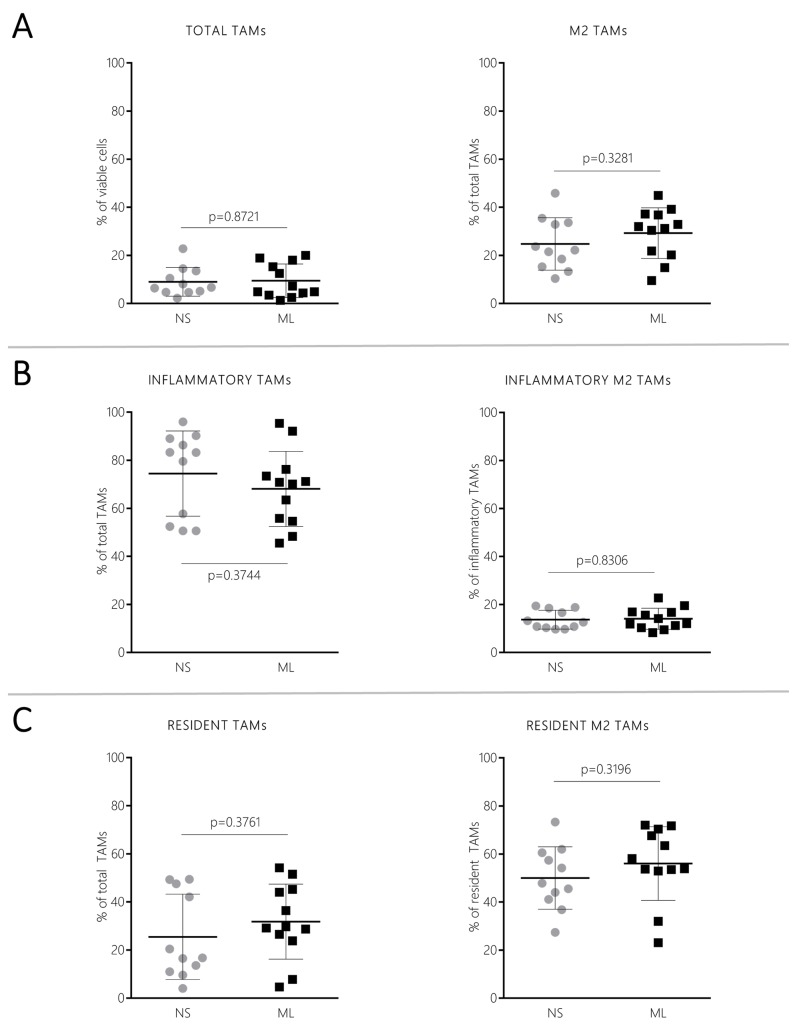
**TAMs infiltrating brain tumors.** Twenty-four mice were inoculated with 5×10^3^NS/ or ML/CT-2A cells (12 mice per group). 19 days after tumor cell inoculation, mice were euthanized and the tumor-infiltrating immune cells were stained for total (A), inflammatory (B) and resident (C) TAMs infiltrating the microenvironment of NS/ and ML/CT-2A tumors (12 mice per group). The staining failed in one NS/CT-2A tumor-bearing mouse. No significant differences were found in TAMs between NS/ and ML/CT-2A tumors. Left panels, total amounts. Right panels, M2 subpopulations. NS, NS/CT-2A tumors; ML, ML/CT-2A tumors; TAMs, tumor-associated microglia/macrophages.

**Fig. 4.**
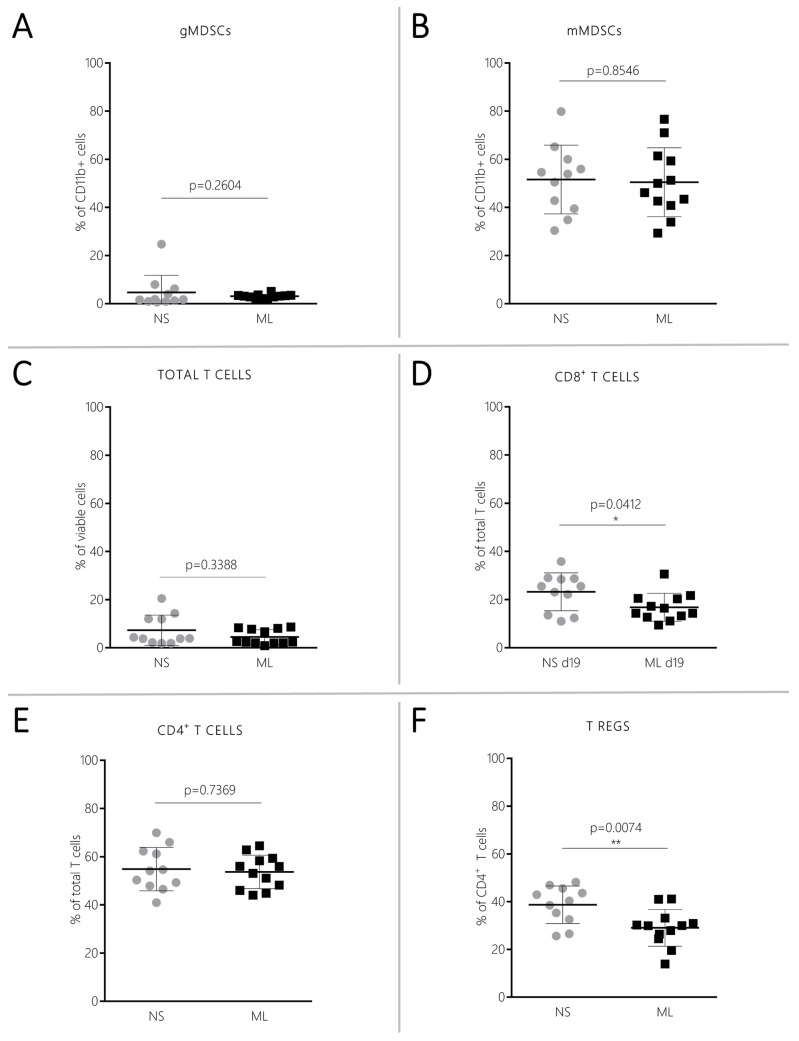
**MDSCs and T cells infiltrating brain tumors.** Twenty-four mice were inoculated with 5×10^3^NS/ or ML/CT-2A cells (12 mice per group). 19 days after tumor cells inoculation, mice were euthanized and the tumor-infiltrating immune cells were stained for gMDSCs (A), mMDSCs (B), total T cells (C), CD8^+^ T cells (D), CD4^+^ T cells (E) and Tregs (F). The staining failed in one NS/CT-2A tumor-bearing mouse. NS/CT-2A mice showed a significantly higher percentage of Tregs and CD8+ T cells compared to ML/CT-2A tumors. NS, NS/CT-2A tumors; ML, ML/CT-2A tumors; g and mMDSCs, granulocytic and monocytic myeloid-derived suppressor cells; CD, cluster of differentiation; Tregs, regulatory T cells.

To investigate whether NS/CT-2A and ML/CT-2A tumors were able to influence the local immune microenvironment differently, brain-infiltrating immune cells were isolated from 24 mice inoculated with either NS/CT-2A or ML/CT-2A cells. The isolation procedure failed in one of the 12 mice inoculated with NS/CT-2A cells. Five to 12×10^6^ immune cells were isolated from each brain and 2.5 to 4×10^6^ cells were used for tumor-associated microglia/macrophages (TAMs), myeloid-derived suppressor cells (MDSCs) and T cells staining. There were no significant differences in innate immune cell infiltration between NS/CT-2A and ML/CT-2A tumors. The total TAM population and its M2 subset showed comparable values in NS/CT-2A and ML/CT-2A tumors (*P*=0.8271 and *P*=0.3281, respectively; Fig. 3A). The largest fraction of TAMs was recruited from blood circulation (Ly6C^+^ TAMs, approximately 70% of total TAMs) while a smaller fraction was due to resident cells (Ly6C^−^ TAMs, approximately 70% of total TAMs; Fig. 3B–C). However, when comparing NS/CT-2A and ML/CT-2A tumors, no significant differences were found in total amount of resident and inflammatory TAMs (*P*=0.3761 and *P*=0.3744, respectively; Fig. 3B–C, left panels) or in their M2 subsets (*P*=0.3196 and *P*=0.8306, respectively; Fig. 3B–C, right panels). Among MDSCs, the more immune-suppressive monocytic subtype (mMDSC) was largely more abundant than the granulocytic subtype (gMDSC); however, no significant differences in the MDSC populations were found between NS/CT-2A and ML/CT-2A tumors (*P*=0.2604 for gMDSCs and *P*=0.8546 for mMDSCs; Fig. 4A–B). In the context of the adaptive immune system, the total amount of T cells did not show significant difference between NS/CT-2A and ML/CT-2A tumors (*P*=0.4338; Fig. 4C). Cytotoxic CD8^+^ T cells were more abundant in NS/ than in ML/CT-2A tumors (23.2±7.5 versus 16.8±5.6% of T cells, *P*=0.0412, respectively; Fig. 4D). However, despite the fact that CD4^+^ T cells appeared in similar amounts in the two tumor types (*P*=0.7369; Fig. 4E), the immune-suppressive regulatory T cells (Tregs) were more abundant in NS/CT-2A than in ML/CT-2A tumors and this difference reached a high level of statistical significance (38.7±7.5 versus 29.0±7.4% of CD4^+^ T cells, *P*=0.0074, respectively; Fig. 4F).

### CT-2A NS drive splenocytes towards a more immunosuppressed phenotype

**Fig. 5.**
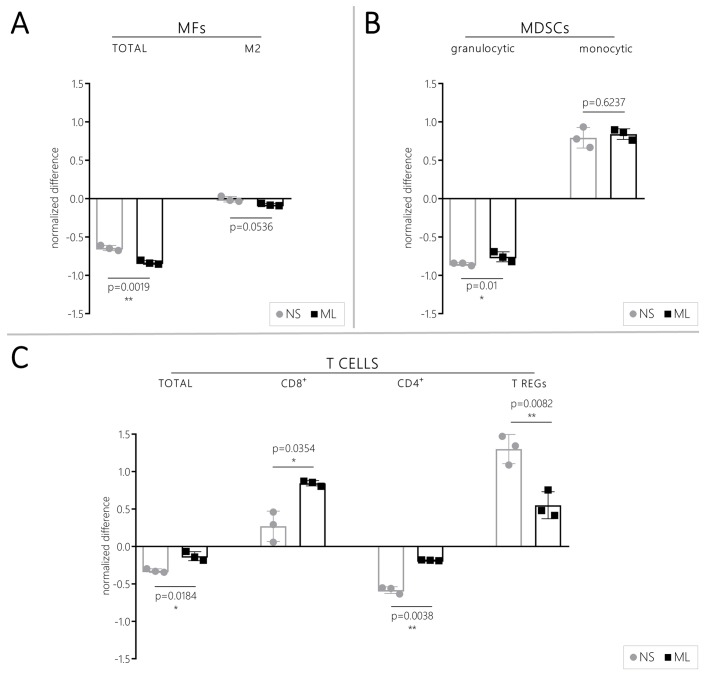
***In vitro* immune effect of CT-2A cells.** Modification of MFs (A), MDSCs (B) and T cells (C) subpopulations of splenocytes after 48-h co-culture with NS/ and ML/CT-2A cells. Compared to ML/CT-2A-splenocytes co-cultures, NS/CT-2A-splenocytes co-cultures showed higher amount of MF and Tregs and significantly lower amount of gMDSCs, Total T cells, CD8+ T cells and CD4+ T cells. Values are expressed as normalized difference between the study conditions (NS/ or ML/CT-2A cells and splenocytes in NS or ML medium, respectively) and the corresponding control conditions (splenocytes in NS or ML medium, respectively). NS, NS/CT-2A cells; ML, ML/CT-2A cells; MF, macrophages; MDSCs, myeloid-derived suppressor cells; CD, cluster of differentiation; Tregs, regulatory T cells.

In order to provide a better understanding of *in vivo* results, we co-cultured CT-2A cells (in either ML or NS conditions) with naive splenocytes (obtained from three different mice), and we analyzed the immune-modulatory effects exerted by CT-2A cells on splenocytes *in vitro*. In this setting, NS/CT-2A and ML/CT-2A cells were able to modulate the immune subpopulation of splenocytes to a different extent. Compared to the control conditions, both NS/ and ML/CT-2A cells induced a reduction of macrophages (MFs, Fig. 5A). However, such a reduction was more pronounced when ML/CT-2A cells were present: flow-cytometry demonstrated significantly higher proportion of total MFs (*P*=0.0019) and a strong trend towards a higher proportion of M2 MFs (*P*=0.0536) in splenocytes-NS/CT-2A co-cultures than in splenocytes-ML/CT-2A co-cultures. Both NS/CT-2A and ML/CT-2A cells induced a decrease of gMDSCs and increase of mMDSCs (Fig. 5B) compared to control conditions. No difference in mMDSCs was found between NS/CT-2A and ML/CT-2A cells (*P*=0.6237), while gMDSCs were slightly more abundant in the presence of NS/CT-2A cells (*P*=0.01). Significant results were observed in the context of the adaptive immune system (Fig. 5C). Compared to control conditions, the presence of CT-2A cells induced a decrease of total T cells and CD4^+^ T cells. In both cases, this decrease was significantly more pronounced when NS/CT-2A cells were present (*P*=0.0184 and *P*=0.0038, respectively). Conversely, CD8^+^ T cells and regulatory T cells were both increased in co-cultures compared to control conditions; however, CD8^+^ T cells proportion was higher for ML/CT-2A cells (*P*=0.0354) while Tregs proportion was higher for NS/CT-2A cells (*P*=0.0082).

### No significant differences in the molecular composition or in vascular permeability were found in CT-2A NS- and ML-derived tumors

**Fig. 6.**
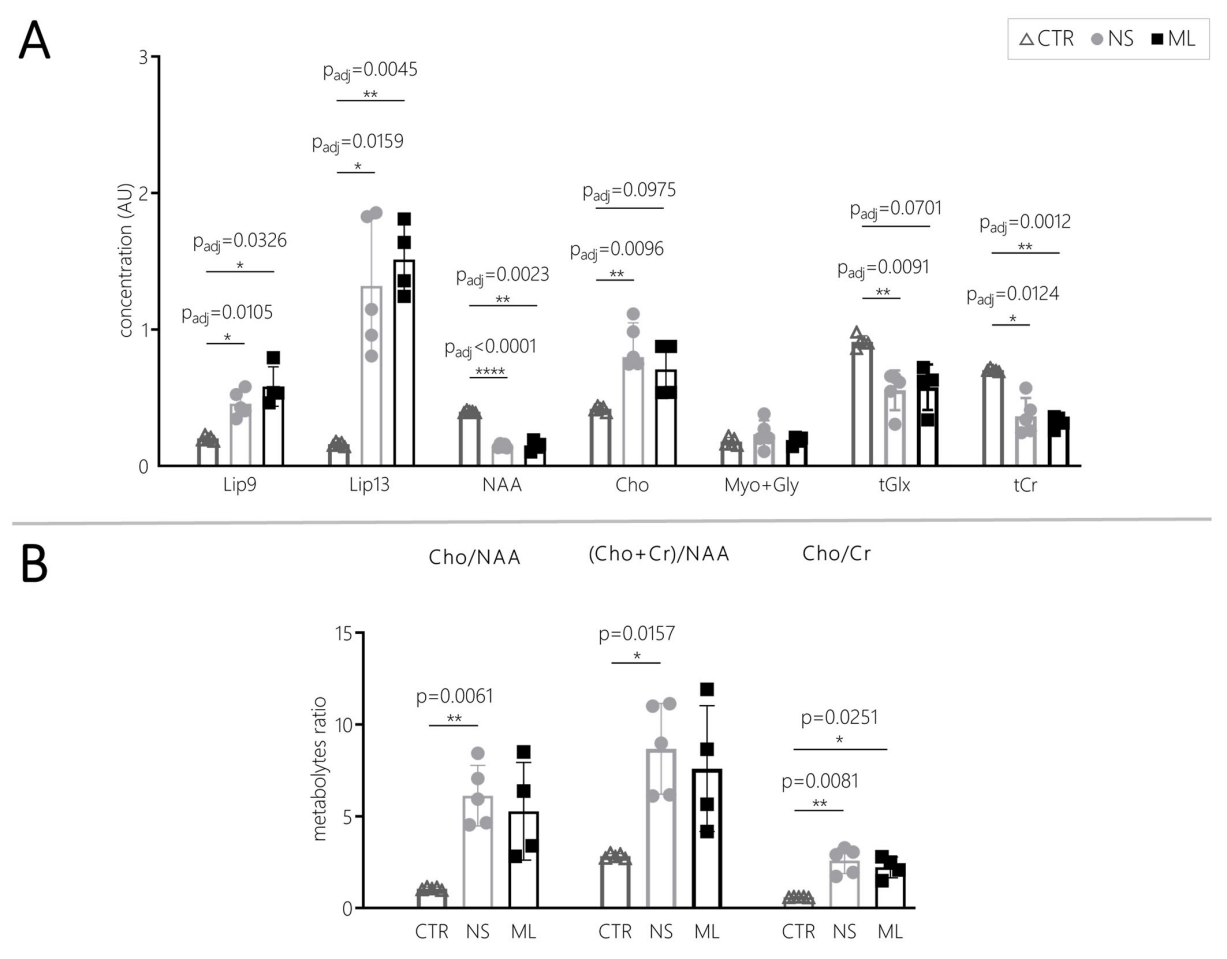
**MR spectroscopy of CT-2A tumors.** Ten mice were inoculated with 5×10^3^NS/ or ML/CT-2A cells (five mice per group). 19 days after tumor cells inoculation, tumors were analyzed with MRS for metabolites' content (A). Both NS/ and ML/CT-2A MRS spectra resembled that of human HGG (Hsu et al., 2004; Kousi et al., 2012); however, no significant differences were found between the two models. We also analyzed metabolites ratios known to be increased in human HGG (Hsu et al., 2004; Kousi et al., 2012). Despite not reaching statistical significance when comparing ML/ and NS/CT-2A tumors, all these ratios were in favor of a higher malignancy of the latter; furthermore, all these three metabolite ratio were significantly higher in NS/CT-2A tumors when compared with control brain whereas only the increase of Cho/Cr reached statistical significance when comparing normal brain and ML/CT-2A tumors (B). NS, NS/CT-2A tumors; ML, ML/CT-2A tumors; CTR, controls; Lip0.9, lipids at 0.90 ppm; Lip1.3, lipids at 1.30 ppm; NAA, N-acetylaspartate; Cho, choline and other trimethylamine-containing compounds; Cr, creatine and phosphocreatine; Myo+Gly, glycine and myo-inositol; Glx, glutamate and glutamine.

We performed magnetic resonance spectroscopy (MRS) in order to evaluate the biochemical changes in CT-2A tumors. Five NS/ and five ML/CT-2A tumors were analyzed; however, the quality of the MRS spectrum of one ML/CT-2A tumor was too low quality and such sample was therefore excluded from analysis. The following metabolites' peak were identified: glycine and myo-inositol (Myo+Gly) at 3.55 ppm, choline and other trimethylamine-containing compounds (Cho) at 3.20 ppm, creatine and phosphocreatine (Cr) at 3.03 ppm, glutamate and glutamine (Glx) at 2.35 ppm, N-acetylaspartate (NAA) at 2.02 ppm, and lipids at 1.30 ppm (Lip 1.3) and 0.90 ppm (Lip 0.9). Compared with the normal brain parenchyma, brain tumors showed significantly increased Lip 0.9 (*P*=0.0105 for NS/ and *P*=0.0326 for ML/CT-2A tumors) and Lip 1.3 (*P*=0.0159 for NS/ and *P*=0.0045 for ML/CT-2A tumors) indicating necrosis, decreased NAA (*P*<0.0001 for NS/ and *P*=0.0025 for ML/CT-2A tumors) and Glx (*P*=0.0091 for NS/ and *P*=0.0701 for ML/CT-2A tumors) indicating loss of neurons, increased Cho (*P*=0.0096 for NS/ and *P*=0.0975 for ML/CT-2A tumors) indicating incremented cell proliferation and decreased Cr (*P*=0.00124 for NS/ and *P*=0.0012 for ML/CT-2A tumors) indicating incremented metabolic demands of the tumor tissue (Fig. 6A). These modifications have been already reported in rodents (Tkáč et al., 2003) and humans (Majós et al., 2004; Van Cauter et al., 2014) and are indicative of a high grade tumor of glial origin. We have noticed some variability between metabolite content in individual tumors; however, no significant differences were found in any of these metabolites when comparing NS/CT-2A and ML/CT-2A tumors (Fig. 6A). We also analyzed typically used metabolite ratios such as Cho/NAA, (Cho+Cr)/NAA and Cho/Cr, since previous studies correlated the increase of these values with an increased malignancy of primary brain tumors (Hsu et al., 2004; Kousi et al., 2012). No statistical differences were found between NS/ and ML/CT-2A tumors. However, all these three metabolite ratio were significantly higher in NS/CT-2A tumors when compared with control brain (*P*=0.0061, *P*=0.0157 and *P*=0.0081 for Cho/Naa, Cho+Cr)/NAA and Cho/Cr, respectively) whereas only the increase of Cho/Cr reached statistical significance when comparing normal brain and ML/CT-2A tumors (*P*=0.0251, Fig. 6B). Representative spectra of controls, ML/ and NS/CT-2A tumors can be seen in Fig. S3.

Dynamic contrast enhancement (DCE) magnetic resonance imaging (MRI) was performed to evaluate the permeability of the tumor vessels. Four animals in each group underwent the DCE-MRI scan protocol. Due to technical problems, analysis could only be performed on three ML/ and one NS/CT-2A tumors. Parametric maps (Fig. S4) show that both types of tumors have increased fractional volume, an index of total blood volume. In this late stage of tumor development, k_trans_ and FV values vary across different tumor areas, indicating areas of hyperperfusion and areas of hypoperfusion. The latter, most likely indicating necrotic regions. However, the group size was too small to conclude on group differences in the DCE-MRI data.

## DISCUSSION

In recent years, two factors have been considered important as causes of treatment failure in HGGs: the presence of a population of GSCs with immune-escaping and treatment-resistance capability, and the presence of a highly immunosuppressive local microenvironment hampering an appropriate anti-tumor immune response and reducing the effects of immunotherapeutic strategies. Several preclinical studies addressed the role of GSCs in HGGs; however, most of these studies were based on deriving GSCs from human samples and re-implanting them in immune-deficient xenografts (Audia et al., 2017). The use of immune-deficient models have important limitations, since they obviously do not allow to analyze the interactions between GSCs and the immune microenvironment of HGGs. Performing preclinical research using HGG *in vivo* models with an enriched GSC population and a functional immune system represents a fundamental prerequisite to develop more effective treatments; nevertheless, such models are currently lacking. In the attempt to overcome this limitation, we used NS/CT-2A cells to generate a new HGG model in immunocompetent mice and we performed a comparative characterization of NS/CT-2A tumors and the standard ML/CT-2A tumors.

The NS assay is commonly accepted as the technique of choice to generate HGG primary cultures from HGG patients' samples. This technique is supposed to recreate conditions which are closer to the *in vivo* situation and to maintain/enrich the GSCs population (Gil-Perotín et al., 2013). However, to the best of our knowledge, no study analyzed so far the difference between *in vivo* tumors generated via implantation of the same type of HGG cells cultured in NS or ML. Specifically for the murine HGG cell-line CT-2A, it was not clear whether the increase of GSCs seen in NS culture was limited to the *in vitro* setting or if it could be maintained also after *in vivo* inoculation (Binello et al., 2012). We demonstrated that, compared to their ML counterpart, NS/CT-2A tumors had a significantly higher expression of Nestin and a trend towards a higher expression of CD133. We also showed that NS/CT-2A tumors are more aggressive: the tumor growth was faster, the tumor volumes at endpoints were larger and the survival of tumor-bearing mice was reduced. A variety of markers have been proposed for the identification of GSCs, of which CD133 and Nestin are most often used (Abou-Antoun et al., 2017). Despite conflicting literature results concerning the actual prognostic values of stemness markers in HGGs, a recent systematic review and meta-analysis showed that a higher expression of Nestin and CD133 in HGG patients is associated with a worse prognosis (Wu et al., 2015). In the studies included in such systematic review, the amount of CD133+ and Nestin+ cells in human gliomas ranged between 0.5 and over 82% and between 1–100%, respectively (Arai et al., 2012; Hatanpaa et al., 2014; Kase et al., 2013; Kim et al., 2011; Melguizo et al., 2012; Wan et al., 2011). Compared to the standard CT-2A model, in our NS/CT-2A model the amount of Nestin-positive cells was increased from 35.5 to 72.7% and the amount of CD133-positive cells was increased from 1.9 to 4.0%. Therefore, the amount of GSCs in NS/CT-2A tumors was increased compared to its ML counterpart but still within physiological levels. Of note, it has been proved not only GSCs but also other cell types (such as endothelial cells or reactive glia) can express Nestin (Arai et al., 2012; Krum and Rosenstein, 1999; Lin et al., 1995). By using CT-2A cells transfected with a reporter gene, we could have discriminated between tumorous and non-tumorous Nestin-positive cells. However, the pathological assessment of Nestin expression in human samples is performed on all the cells within the tumor mass (for obvious reasons, since human tumor cells do not express a reporter gene). Therefore, it is this total expression of Nestin within the tumor (and not only its tumorous component) that has a prognostic value in patients (Wu et al., 2015). For these reasons, in the present study we decided to follow the same approach currently used in the clinic and to refer to the total amount of Nestin-positive cells within the tumors for the assessment of Nestin expression. Taken together, these data confirm the high translational impact of our mouse model and proves that it represents a valuable platform for the development of innovative treatments directed against GSCs.

Vascular proliferation is a key feature in HGGs and in particular in GBMs (Urbańska et al., 2014). Due to the aberrant neoangiogenesis seen in these neoplasms, the tumor microenvironment remains hypoxic (Audia et al., 2017). Several studies indicate hypoxia as a fundamental factor in maintaining GSCs; in turn, GSCs have a role in the formation of dysfunctional vessels, further increasing hypoxia (Audia et al., 2017; Roos et al., 2017). In the present study, we demonstrated a strong trend towards an increased vascularity in NS/CT-2A tumors that could also be linked to their increased GSCs population. This broadens the possible applications of the NS/CT-2A tumor model to study treatment strategies aiming at normalizing the tumor vasculature and reducing hypoxia.

The analysis of H&E and GFAP sections of NS/CT-2A tumors demonstrated the presence of malignant and highly proliferative brain neoplasms of glial origin (Fig. S2) neuropathologically behaving like a high-grade anaplastic astrocytoma, probably in transition to a GBM in the light of the high mitotic activity. However, NS/CT-2A tumors lacked some features in order to be canonically categorized as GBM, namely microvascular proliferation and pseudo-palisading necrosis. Both these features were seen in the study of Martinez-Murillo and colleagues, which characterized the standard ML/CT-2A tumors (Martinez-Murillo and Martinez, 2007). Nevertheless, two important findings should be taken into account. First, despite it not being possible to demonstrate microvascular proliferation in our model, we showed (as already mentioned) an increased microvascular density (CD31+ structures) in NS/CT-2A tumors compared to their ML counterpart. Second, Martinez-Murillo and colleagues evaluated the presence of necrosis at a later time point compared to ours; therefore, it is possible that such feature would also develop in our model at a more advanced stage.

Despite the large amount of research investigating the role of the immune system in glioma progression, the relationship between the HGG's immune microenvironment and GSCs is not fully understood yet (Audia et al., 2017). As mentioned previously, this is mainly because most of the studies concerning GSCs have been performed using xenograft models. The literature shows that GSCs modulate the innate and adaptive immune response via several mechanisms such as evasion of T-cell recognition, inhibition of T-cell activation, Treg expansion, recruitment of TAMs and their M2-polarization (Audia et al., 2017; Marincola et al., 2000; Roos et al., 2017; Wu et al., 2010). It is also interesting to note that Tregs can promote GCSs’ expansion and therefore their immune suppressive role (Fukumori et al., 2003; Stillman et al., 2006). However, these data were mainly gathered from the analysis of human HGG samples and from *in vitro* HGG studies, or from research performed for other tumor types. As already mentioned, up until now a translational murine model specifically characterized to simultaneously address the biology of GSCs and immune cells, and to develop treatment strategies aimed at blocking their reciprocal support, was still missing. In the present study, we analyzed the effect of NS/ and ML/CT-2A cells on immune cells both *in vivo* and *in vitro*. *In vivo*, NS/CT-2A tumors induced an increased amount of Tregs compared to ML tumors, and this increase reached a high level of significance (*P*=0.0074). Nevertheless, NS/CT-2A tumors also showed a higher amount of CD8^+^ T cells. Despite *in vitro* studies showing that Tregs have an influence on CD8^+^ T cells proliferation, *in vivo* studies demonstrate that the inhibition of CD8+ T cells can also be achieved via impairment of their cytotoxic functions without affecting their expansion (Schmidt et al., 2012). Interestingly, this has been proven not only in cancer but also in auto-immune response models (Mempel et al., 2006). In this regard, we could also speculate that the effector cells in our NS/CT-2A model are rendered anergic due to the presence of a high amount of both Tregs and GSCs, and the absolute number of cytotoxic T cells is not so relevant. No significant differences in the myeloid compartment have been found between NS/CT-2A and ML/CT-2A tumors. In both types of tumors, a high amount of immunosuppressive cells such as M2 TAMs and mMDSCs were present. Of note, the differences in the immune phenotypes of NS and ML/CT-2A tumors were significant but not dramatic. This result was expected since these two models, even if different from the immunological point of view, are based on the same cell-line.

In CT-2A/splenocytes co-cultures, NS/CT-2A cells were able to drive both the innate and the adaptive immune system towards a more immune-suppressive status. In particular, NS/CT-2A cells induced a higher proliferation of total MF and their M2 sub-fraction, a higher proliferation of Tregs and a lower proliferation of total T cells, CD4^+^ T cells and CD8^+^ T cells compared to ML/CT-2A cells.

In conclusion, these results show that NS/CT-2A are able to modulate the immune microenvironment towards a more suppressive state both *in vitro* and *in vivo*. In patients, elevated levels of Tregs and GSCs correlate with a poorer survival rate (Wu et al., 2015; Yue et al., 2014): the higher levels of Tregs and GSCs in the NS/CT-2A model increase its translational value as a model for HGG. The model is therefore suitable for studying the complex interactions between GSCs and the immune system and for the preclinical testing of innovative therapeutic agents directed against these targets.

This study also presents limitations. First, we limited ourselves to only one cell-line, namely CT-2A, as this specific type of murine HGG cells could be cultured in NS in order to increase the number of GSCs. In future, we can test other murine HGG cell-lines in NS cultures to expand our research platform and our understanding of the relationship between the immune system and GSCs. Second, the flow-cytometry panel used for T cells did not include markers of activation or exhaustion. Such markers can provide useful information concerning the immune phenotype of the tumor microenvironment; however, we felt that this was beyond the scope of this paper. Third, it would be interesting not only to study the number and subtype of brain infiltrating immune cells, as we did by flow-cytometry, but also to investigate their spatial distribution within the tumor–immune microenvironment. Fourth, while the longitudinal anatomical *in vivo* MR study showed clear differences between ML/ and NS/CT-2A tumors, near end-point MRS and DCE could confirm the HGG nature of these neoplasms but they failed in demonstrating significant differences (probably, due to the large variability of the samples). However, longitudinal MRS and DCE-MRI experiments might have shown differences at early time points.

### Conclusion

In this study, we demonstrated that NS/CT-2A tumors grew faster, sustained a higher proliferation of GSCs and showed an increased immunosuppression compared to standard ML/CT-2A tumors. For this reason, the NS/CT-2A tumor model could represent a suitable and high translational research platform for the study of innovative treatments against HGGs, in particular when aiming at eliminating GSCs and reversing the tumor-induced immunosuppression. We believe that the next generation of preclinical studies must be performed in multiple HGG models, including patient-derived xenografts, genetically engineered models and our model. This will strongly potentiate the translational impact of such studies and accelerate the development of effective therapies for HGG patients.

## MATERIALS AND METHODS

### Tumor cell cultures

CT-2A cells were provided by Prof. Thomas Seyfried (Boston College, Boston, MA, USA) (Seyfried et al., 1992). Cells were incubated at 37°C in humidified air with 5% CO_2_. ML/CT-2A cells were cultured in Dulbecco's Modified Eagle Medium (DMEM; Thermo Fisher Scientific), supplemented with 10% heat-inactivated fetal calf serum (FCS; Thermo Fisher Scientific) (Seyfried et al., 1992). To generate NS/CT-2A cells, we adapted a previously published protocol (Binello et al., 2012). Briefly, confluent ML were enzymatically dissociated (Stempro Accutase, Thermo Fisher Scientific) and cells were plated at 1×10^5^ cells/ml in DMEM/Nutrient Mixture F-12 (DMEM/F-12; Thermo Fisher Scientific) supplemented with 20 ng/ml epidermal growth factor (EGF; Thermo Fisher Scientific), 20 ng/ml fibroblast growth factor (FGF; Thermo Fisher Scientific) and 2% B27 supplement (Thermo Fisher Scientific). Six days after plating, NS were collected, enzymatically dissociated and re-plated at the same initial concentration. Two and 8 days after the start of the NS culture, the medium was supplemented with 20 ng/ml EGF and FGF. Eleven days after the start of the culture, NS were collected, enzymatically dissociated and prepared for further applications.

### Animals and *in vivo* generation of brain tumors

Female adult (12–14 weeks old) C57BL/6J mice (Envigo) were used for this study. CT-2A cells were injected intracranially in C57BL/6J mice following a procedure adapted from a previous publication (Koks et al., 2015). Mice were anesthetized via intraperitoneal (IP) injection of 6 μl/g body weight of a mixture of 18.75 mg/ml ketamine (Pfizer) and 0.125% xylazine (Bayer). By means of a stereotactic frame, 5×10^3^ CT-2A cells suspended in 4 μl of DMEM/F-12 were inoculated 2.5 mm lateral from the midline, 0.5 mm anterior to the bregma and 2.5 mm below the dura mater with a 26-gauge syringe (Hamilton). The stereotactic inoculation was performed under sterile conditions. Afterwards, the mice were weighed and clinical symptoms were evaluated at least three times per week. Mice were euthanized when they lost 20% of their initial weight they reached grade 3–4 symptoms on a scoring system adapted from a previous publication (Maes et al., 2009). The scoring system included five grades: grade 0, no symptoms; grade 1, mild hemi-paresis (mouse moving slower than normal, with no circling behavior); grade 2, moderate hemi-paresis (mouse moving slower, unstable gait with some oscillations or drops, no circling behavior); grade 3, hunched posture, severe hemi-paresis (constant circling behavior, frequent drops, inability to move) or both; grade 4, moribund mouse.

### Ethics approval

All animal experiments were approved by the bioethics committee of the Katholieke Universiteit Leuven (project 089/2015) which follows the European Directive 2010/63/EU.

### Histology and immunohistochemistry

Nineteen days after tumor inoculation, mice were anesthetized with 50 mg/kg Pentobarbital (Lundbeck) IP and a transcardial perfusion with phosphate-buffered saline (PBS) followed by 4% paraformaldehyde (PFA, VWR Chemicals) was performed (Maes et al., 2009). After harvesting, brains were submerged in 4% PFA for 48 h at 4°C, washed and processed for paraffin embedding (HistoStar™ Embedding Workstation). Sections of 4 µm thickness obtained from the paraffin-embedded tissues (Thermo Fisher Scientific, Microm HM355S microtome) were mounted on Superfrost™ Plus Adhesion slides (Thermo Fisher Scientific) and routinely stained with H&E (Diapath #C0302 and #C0362) for histopathological examination. Images were acquired on a Zeiss AxioScan Z1 using a ×20 objective and the software ZEN 2 (Zeiss). Mitotic figures were manually counted in 10 HPF (diameter of each field: 0.4 mm).

For fluorescence IHC, the following antibodies were used to detect the respective proteins: anti-CD133 (rabbit, 1:600, Abcam, ab19898), anti-CD31 (rat, 1:300, Dianova, SZ31), anti-Nestin (rabbit, 1:1000, Sigma-Aldrich, HPA026111). The PerkinElmer Opal 4-Color Manual IHC Kit (PerkinElmer, NEL810001KT) was used to amplify the tyramide signal according to the manufacturer's protocol. The Envision+/HRP goat anti-rabbit (Dako Envision+ Single Reagents, K4003) was used to induce the secondary-HRP of the rabbit primary antibodies and the proteins were detected using the OPAL 570 reagent. Rat primary antibodies were incubated overnight at +4°C in TNB buffer (PerkinElmer, NEL705A001KT) with anti-donkey biotin (1:200, Jackson ImmunoResearch, 711-065-152), and Streptavidin-HRP Conjugate (1:100, PerkinElmer, NEL750001EA) was used for introduction of the secondary-HRP before applying the TSA Cyanine 5 System kit (PerkinElmer, NEL705A001KT) according to the manufacturer's protocol. Digital images were digitally acquired as described above. CD31-stained micrographs were analyzed with the software ImageJ (http://rsb.info.nih.gov/ij). For each section, the tumor mass was manually delineated and the same color threshold was applied to all samples in order to discriminate between CD31-positive and CD31-negative areas. The CD31-positive tumor area was automatically calculated. Nestin- and CD133-stained micrographs were analyzed with the software QuPath (Bankhead et al., 2017). After manually delineating the tumor mass on each section, an automatic cell detection was performed in the DAPI channel. Ten Nestin- or CD133-positive and ten Nestin- or CD133-negative cells were manually selected in one random sample and used to create a ‘cell classifier’ using all the 55 given parameters (for the full list of these parameters, see Table S1). The classifier was applied to all samples and the number of positive cells was automatically calculated.

For bright field IHC, the following antibody was used to detect the respective protein: anti-GFAP (rabbit, 1:300, DAKO, Z0334, polyclonal, Lot 20040599, 2.9 g/l) in TNB buffer (TSA blocking reagent, Perkin Elmer, FP1020). Tissue sections were deparaffinized and hydrated in distilled water. The sections were fixed for 10 min in 10% neutral buffered formalin (NBF) solution, (Sigma-Aldrich, HT501320-9.5L), followed by 23 min of heat-induced epitope retrieval (HIER) in AR6 buffer (Perkin Elmer, AR6001KT) using the 2100 Antigen Retriever (Aptum Biologics Ltd). After a cool-down of 15 min in miliQ water, the endogenous peroxidase activity of the samples was blocked by a 20 min incubation in 0.3% hydrogen peroxide in methanol. The tissue was then blocked for 30 min using a blocking buffer {TBS with 1% BSA [VWR, 22013, Bovine Serum Albumin (BSA), fraction V, Biotium (50 g)]} with 10% normal goat serum (Invitrogen, 10000C) and the sections were incubated for 30 min at room temperature with the antibody solution. For introduction of the secondary-HRP the Envision+/HRP goat anti-rabbit (Dako Envision+ Single Reagents, HRP, Rabbit, Code K4003) was used and samples were incubated at room temperature for 45 min. For the final visualization of the protein the ImmPACT™ DAB peroxidase substrate kit (Vector Labs, SK-4105) was used according to the manufacturer's protocol. The incubation time was 15 min at room temperature. Samples were then counterstained with Hematoxylin solution (Diapath #C0302) and mounted with D.P.X. Neutral mounting medium (Sigma-Aldrich, 317616). Digital images were acquired as described above.

### Tumor-infiltrating immune cells

Nineteen days after tumor inoculation, mice were anesthetized and PBS-perfused as previously described. The brains were harvested and mechanically disrupted using a scalpel. After enzymatic digestion with 1 mg/ml Collagenase I (Thermo Fisher Scientific), 2 mg/ml Dispase I (Thermo Fisher Scientific) and 100 µg/ml DNase I (Sigma-Aldrich), brain tissue was passed through a 70 µm cell strainer (Falcon). Brain-infiltrating immune cells were then isolated by means of a 25% Percoll gradient following a protocol adapted from previous publications (Juan et al., 2012; Pino and Cardona, 2011). Immediately after isolation, immune cells were stained for flow-cytometry. Staining of TAMs, MDSCs and T cells were performed. The antibody panels for TAMs/MDSCs and T cells were adapted from previous publications (Cazareth et al., 2014; Okuneva et al., 2015; Szulzewsky et al., 2015; Vindevogel et al., 2016). Samples were acquired using a BD FACSCanto™ II (BD Bioscience) and results were analyzed with the software FlowJo v.10 (FlowJo LLC). Viable cells were selected based on Fixable Viability Dye (FVD) eFluor780 (Thermo Fisher Scientific) negativity. Total TAMs were identified as CD45^high^CD11b^+^Ly6G^−^F4/80^+^. Cells Ly6C^+^ or Ly6C^−^ were considered as inflammatory and resident TAMs, respectively (Cazareth et al., 2014). TAMs also positive for CD206 were considered alternatively-activated (M2) TAMs. Granulocytic and monocytic MDSCs (gMDSCs and mMDSCs) were identified as CD11b^+^Ly6G^+^Ly6C^−^ and CD11b^+^Ly6C^+^ cells, respectively. Among total T cells (CD45^+^CD3^+^), CD4^+^ and CD8^+^ populations were identified. FoxP3-positive CD4^+^ T cells were considered regulatory T cells (Tregs). Details of the antibodies and the gating strategies are available in Fig. S5.

### *In vitro* co-cultures of splenocytes and CT-2A cells

Naïve mice were euthanized by cervical dislocation. The abdomen was opened under sterile conditions and the spleen was isolated. The spleen was minced and passed through a 70 µm cell strainer (Falcon) and the splenocytes were incubated with red-cell lysis buffer (Invitrogen) to eliminate erythrocytes. Fully formed CT-2A NS and confluent ML, cultured as previously described, were enzymatically dissociated and then co-cultured with splenocytes. A total of 6×10^6^ splenocytes were co-cultured with 1.5×10^6^ NS/CT-2A cells in NS medium or with 1.5×10^6^ ML/CT-2A cells in ML medium (study conditions). As a control condition, 6×10^6^ splenocytes were cultured with either NS or ML medium without CT-2A cells. After 48 h, cells were enzymatically dissociated, collected, stained and analyzed with flow-cytometry. For MDSCs and T cells, the same protocol used for tumor-infiltrating immune cells was applied. For MF, the TAMs protocol previously described was adapted by removing Ly6C staining (Fig. S5a). Since NS and ML CT-2A cells need different media to grow (DMEM/F12+FGF+EGF+B27 and DMED+FCS, respectively), a difference in the splenocytes sub-populations could be induced by either the different tumor cells or the different media, or both. To discriminate the influence of ML and NS/CT-2A cells from that of ML and NS media, results were calculated as normalized differences between the amount of cells in the study condition and the amount of cells in the respective control condition. As an example, the variation of CD8^+^ T cells was calculated as: [(% of CD8^+^ T cells in the study condition−% of CD8^+^ T cells in the corresponding control condition)/% of CD8^+^ T cells in the control condition].

### Magnetic resonance imaging

MRI was performed on a 7 T Biospec small animal MR system (Biospec 70/30, Bruker BioSpin) equipped with actively shielded gradients (200 mT/m) using a 7 cm linearly-polarized resonator for transmission and an actively-decoupled mouse brain surface coil for receiving (Rapid Biomedical). (Leten et al., 2014) Image analysis was performed with the ParaVision 6.0 software (Bruker BioSpin). Mice were anesthetized with isoflurane (Baxter) at 1.5–2%. During acquisition, the breathing rate and body temperature were monitored (SAII, Stony Brook) and maintained at 60–80/min and 37±1°C, respectively.

Morphological MR imaging was performed 7, 14 and 21 days after tumor cells inoculation. A rapid acquisition relaxation enhancement (RARE) T2-weighted sequence with a RARE factor of eight and a repetition time (TR)/echo time (TE) of 2843.5/35 ms. The field of view was 25×25 mm with a matrix of 256×256 pixels. Twenty-five coronal-oriented slices of 0.5 mm thickness and no slice gap were acquired, covering the whole brain. To avoid inter-slice crosstalk, an interlaced scheme was applied. For the *in vivo* quantification of tumor growth, the tumor area was manually delineated on each slice using the ImageJ software (http://rsb.info.nih.gov/ij). Tumor volume was obtained, in compliance with the Cavalieri's principle, by multiplying the tumor areas of each slice by the slice thickness and summation.

MRS was performed 19 days after tumor cells inoculation. 1H-MR spectra were acquired using a Point REsolved SpectroScopy (PRESS) sequence with a TE of 20 ms, TR of 2000 ms, 320 averages, 3300 Hz spectral width and 2048 sampling points. Axial and sagittal T2-weighted MR images were acquired for the placement of the volume of interest (voxel) within the tumor. A cubic voxel of 2.5×2.5×2.5 or 3.0×3.0×3.0 mm^3^ was placed in the tumor area maximizing the amount of tumor tissue present in the voxel. In the few cases where it was still visible, the needle tract was carefully avoided. The water signal was suppressed with a variable power radio frequency pulse and optimized relaxation delays (VAPOR) suppression scheme. Shimming was performed using MAPSHIM protocol, resulting in a water line width at half height <21 Hz. Spectra were processed using jMRUI v6.0 (www.jmrui.eu), which included Fourier transformation, phase correction and baseline correction (Naressi et al., 2001; Stefan et al., 2009). Remaining water signal was suppressed by using a Hankel Lanczos singular values decomposition (HLSVD) filter (van den Boogaart et al., 1994). Metabolites in the frequency domain data were quantified using the in house-built software SPID (Poullet et al., 2007). This quantification is based on peak integration of frequency ranges of the respective metabolites. Relative quantities of the respective metabolites are expressed in arbitrary units (AU). Naïve age-matched healthy mice were use as controls for MRS and metabolites analysis. In these mice, the MRS voxel was placed in the same position (right striatum) as in tumor-bearing mice.

DCE-MRI was also performed 19 days after tumor cells inoculation. DCE-MRI scans were acquired using a fast low angle shot sequence with the following parameters: echo time (TE)=2.90 ms, repetition time (TR)=83.71 ms, flip angle (FA)=40°, 100 repetitions, temporal resolution=7.199 s, four slices (slice thickness=1 mm, slice gap=0.2 mm), matrix=128×128, spatial resolution=0.234 mm isotropic. A bolus of 100 μl of gadopentetate dimeglumine (Magnevist^®^, Bayer, Leverkusen, Germany) diluted at 0.02 mmol/l was injected via the cava vein of the mice after the tenth repetition of the scan. Before and after acquisition of DCE-MRI, T1 parametric maps were acquired with the same spatial geometry as the dynamic scan through a variable repetition time with a RARE readout (variable TRs=1565, 1842, 2227, 2859 and 5000 ms, rare factor=8, effective TE=40 ms). An in-house written tool was used to average, spatially smooth (0.75 voxels) and temporally smooth (rolling mean with window level of 5) the perfusion images using Python (version 2.7 in Spyder). An input function was drawn around the transversal sinus on the dynamic scan and the TOPPCAT plugin (Patlak algorithm) in ImageJ (http://rsb.info.nih.gov/ij) was used to obtain the ktrans and fractional volume (FV) parametric maps of the perfusion images (hematocrit=0.4) (Barboriak et al., 2004). A brain mask was applied after manual delineation.

### Statistical analysis

Statistical analysis was performed using Prism v.7.0 (GraphPad Software). A two-tailed *P*-value <0.05 was considered significant. For survival data, a log-rank (Mantel-Cox) test was performed and data were expressed as median survival. For flow cytometry, MR and immunohistochemistry, data were tested for normal distribution using D'Agostino-Pearson (if *n*≥8) or Shapiro–Wilk (if *n*<8) test. If data were normally distributed, they were expressed as means and standard deviations, and unpaired *t*-test with Welch's correction was used for comparison. If data were not normally distributed, they were expressed as medians and interquartile ranges, and Mann–Whitney test was used for comparison.

## Supplementary Material

Supplementary information
