# Supplementary material for: CT-2A neurospheres-derived high-grade glioma in mice: a new model to address tumor stem cells and immunosuppression
Source: Biol Open. 2019 Sep 11;8(9):bio044552. doi: 10.1242/bio.044552 (PMC6777368; doi:10.1242/bio.044552)
Supplement: Supplementary information [file biolopen-8-044552-s1.pdf]

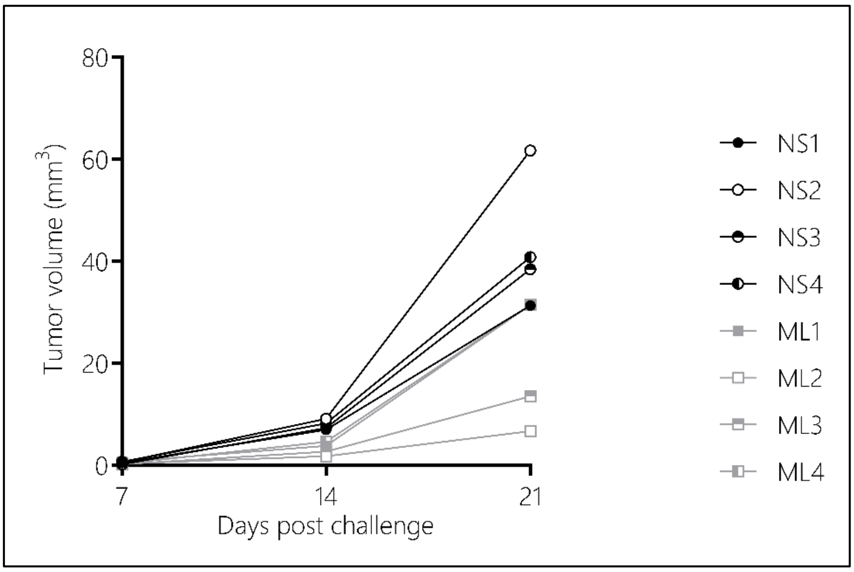

Figure S1. Individual tumor growth curves for NS and ML/CT-2A tumors

Biology Open • Supplementary information

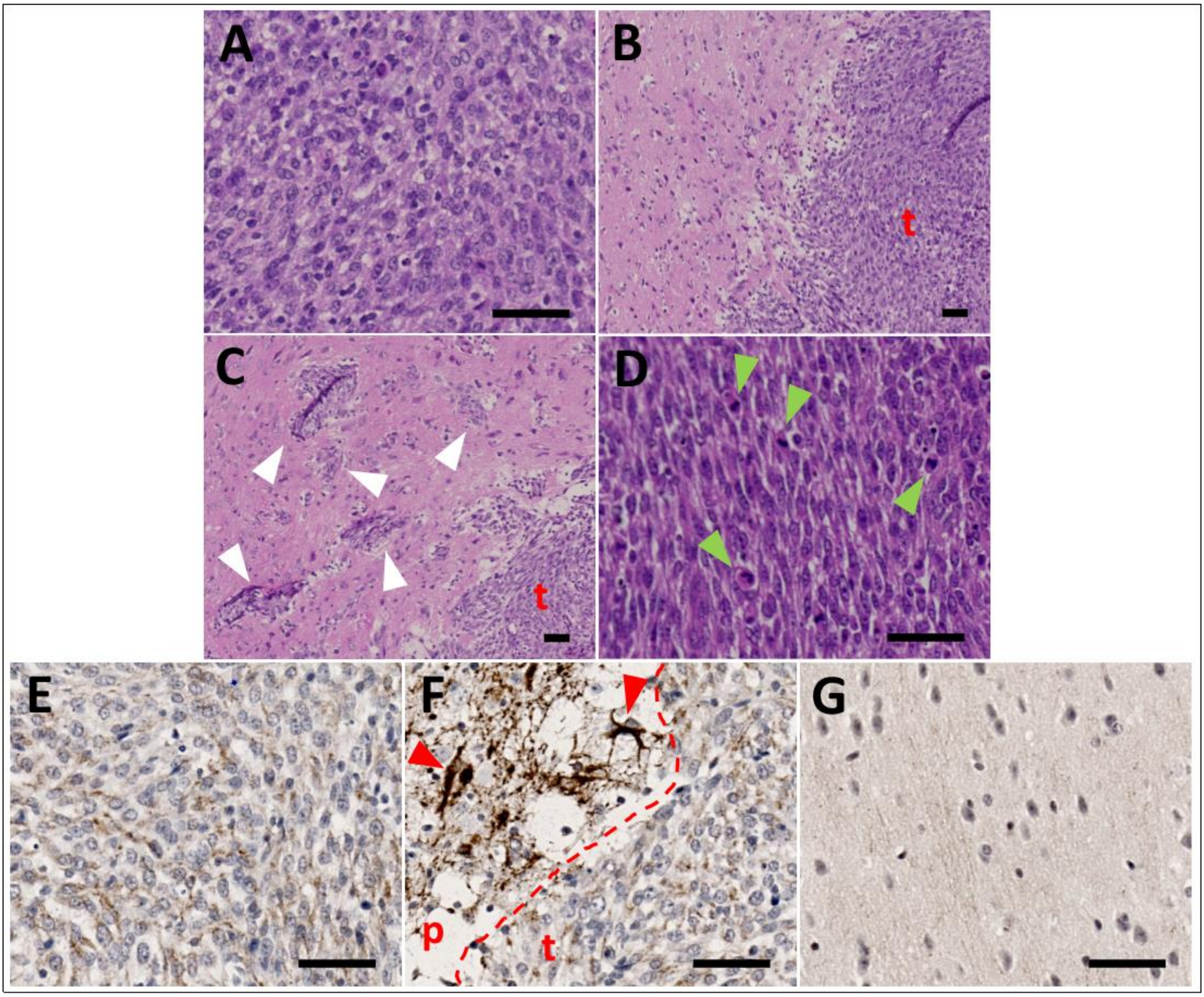

Figure S2. H&E and GFAP stainings of NS/CT-2A tumors. Stainings were performed at day 19 post-tumor cells inoculation. In NS/CT-2A tumor the H&E stainings demonstrated the presence high cellular density (A) and a combination of expansive (B) and invasive (C) growth with the development of satellite lesions (the largest of which are indicated by white arrowheads) at a distance from the main tumor mass. The tumor showed a high proliferation rate, with 21 mitotic figures counted at 10 HPF (diameter of each field: 0.4 mm). Examples of mitotic figures are indicated by green harrowheads in D. NS/CT-2A tumors showed diffuse positivity for GFAP (E). Reactive astrocytes (some of them indicated by red arrowheads) were visible at the tumor margin (F). The contralateral striatum was substantially negative for GFAP (G). Abbreviations: t, tumor mass; p, peritumor area. Dashed line in F represents the limit between the tumor mass and the peritumor area. Scale bars: 50 µm.

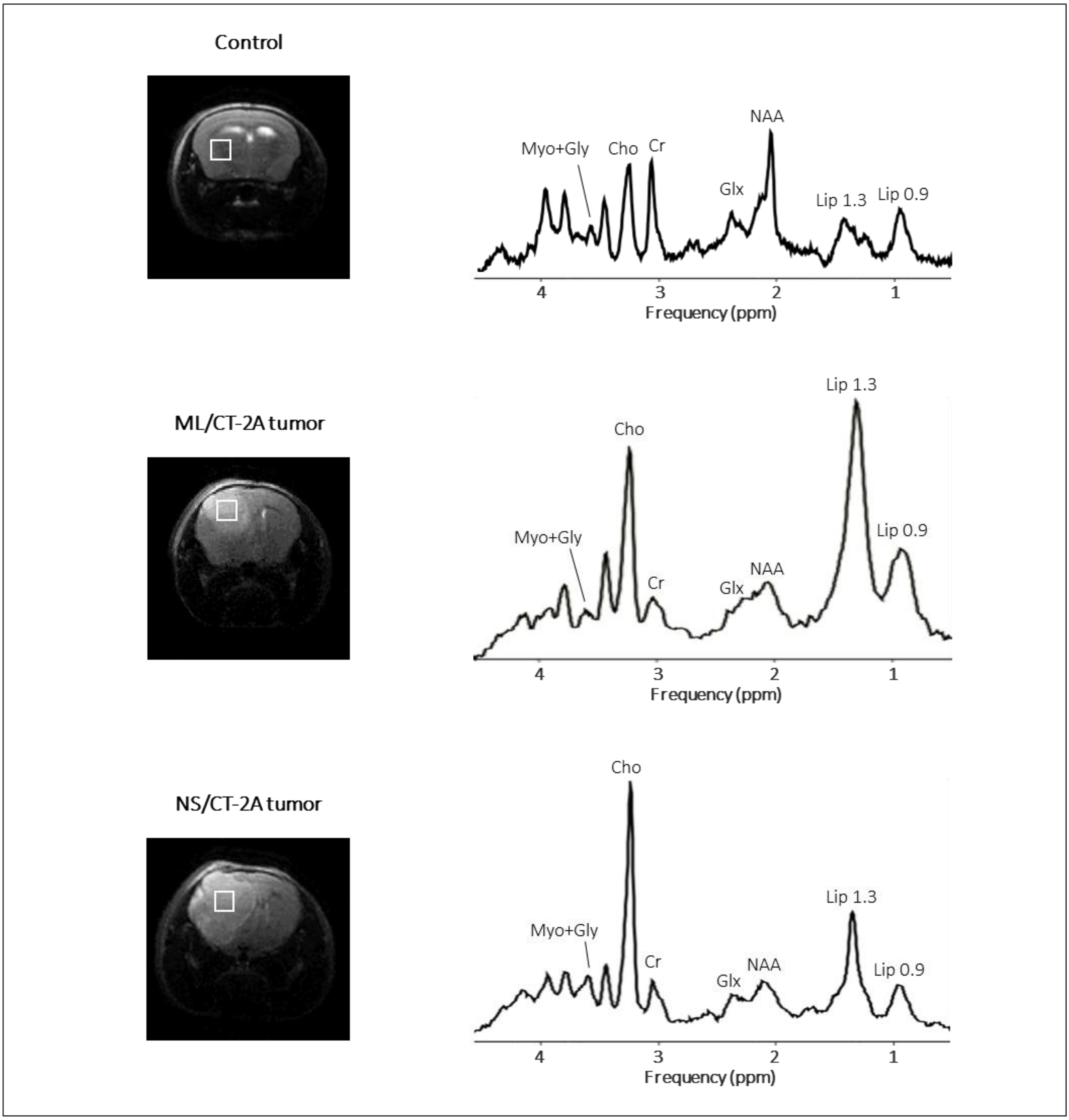

Figure S3. Representative voxel position on axial T2-weighted brain MRI (left panels) and corresponding MRS spectra (right panels) of control healthy mice, ML/CT-2A and NS/CT-2A tumors.

Biology Open • Supplementary information

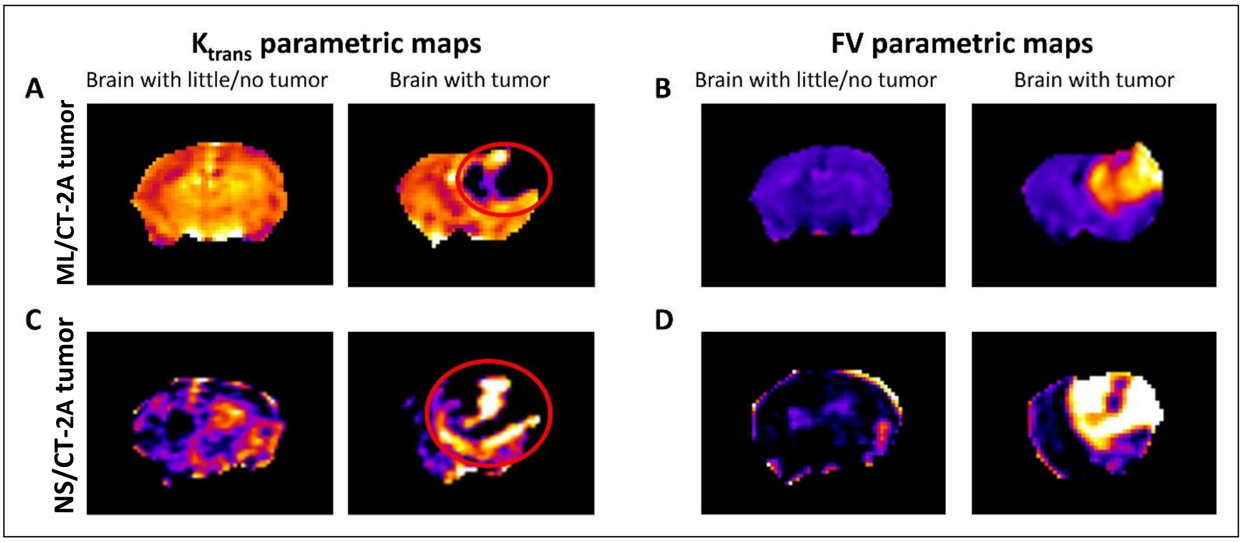

Figure S4. Representative k_{trans} and fractional volume (FV) parametric maps of the brain of mice harboring ML/CT-2A and NS/CT-2A tumors. The left image of the respective panels shows a slice with no (little) tumor (‘control’ region) while the right image of the respective panels shows the slice with maximal tumor size. Tumors are indicated with a red circle on the k_{trans} parametric maps. All tumors in both categories are perfused. Tumors grown from neurospheres are largest as indicated by the tumor spreading over all four slices acquired and by the tumor spreading to both hemisperes of the brain.

Biology Open • Supplementary information

Figure S5a: flow-cytometry gating strategy for in-vivo TAMs (full and dashed lines) and in-vitro MFs (full lines only)

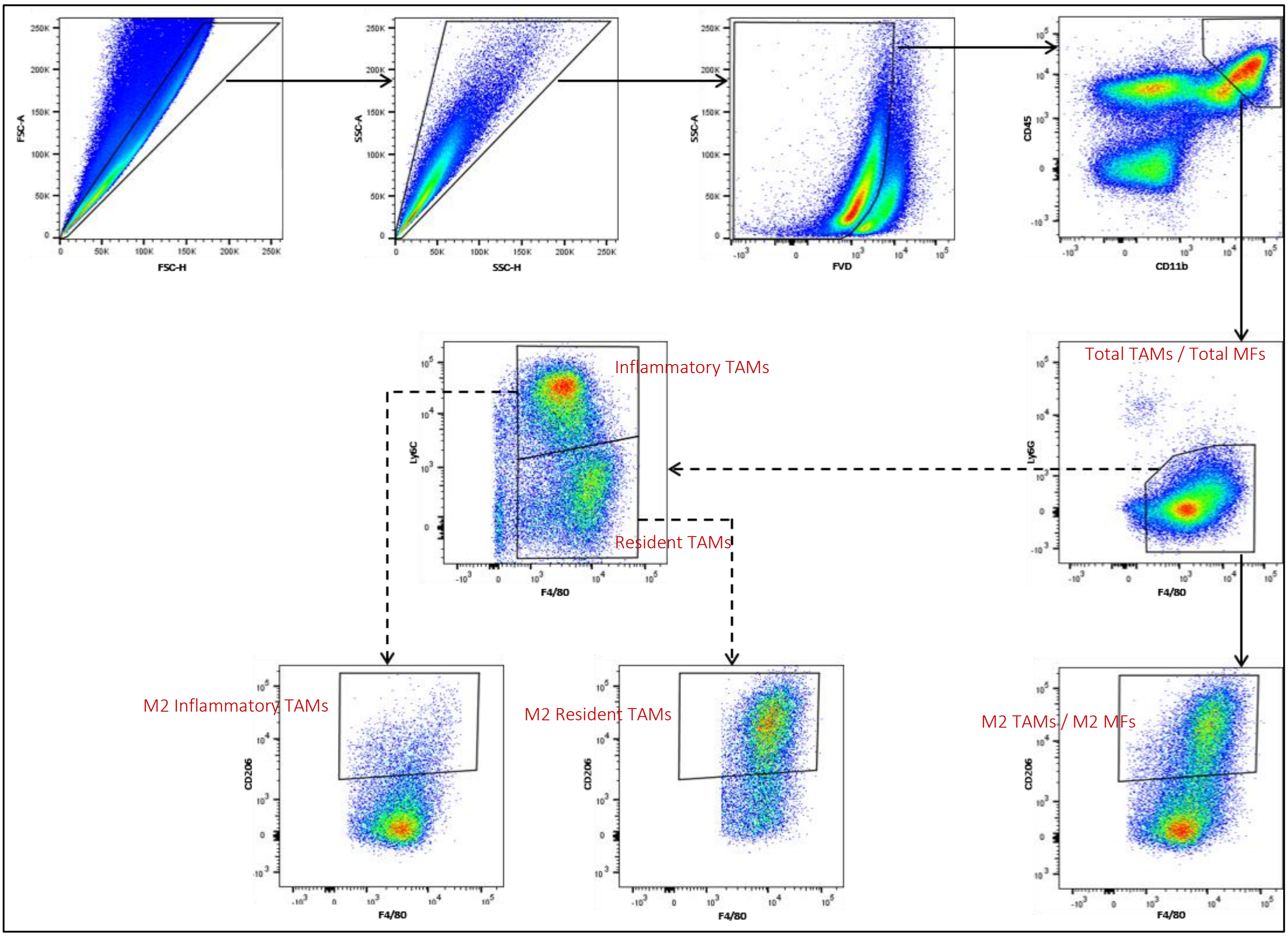

Figure S5b: flow-cytometry gating strategy for in-vivo and in-vitro MDSCs

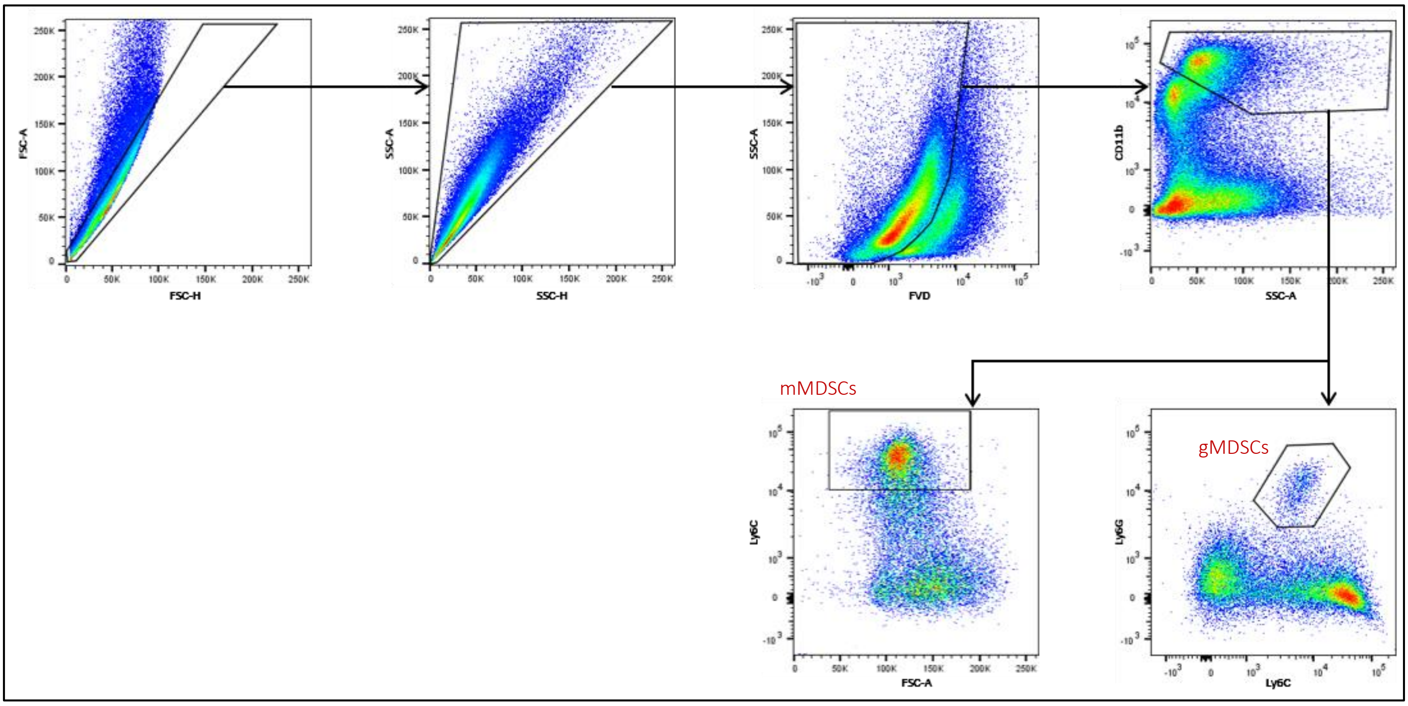

Figure S5c: flow-cytometry gating strategy for in-vivo and in-vitro T cells

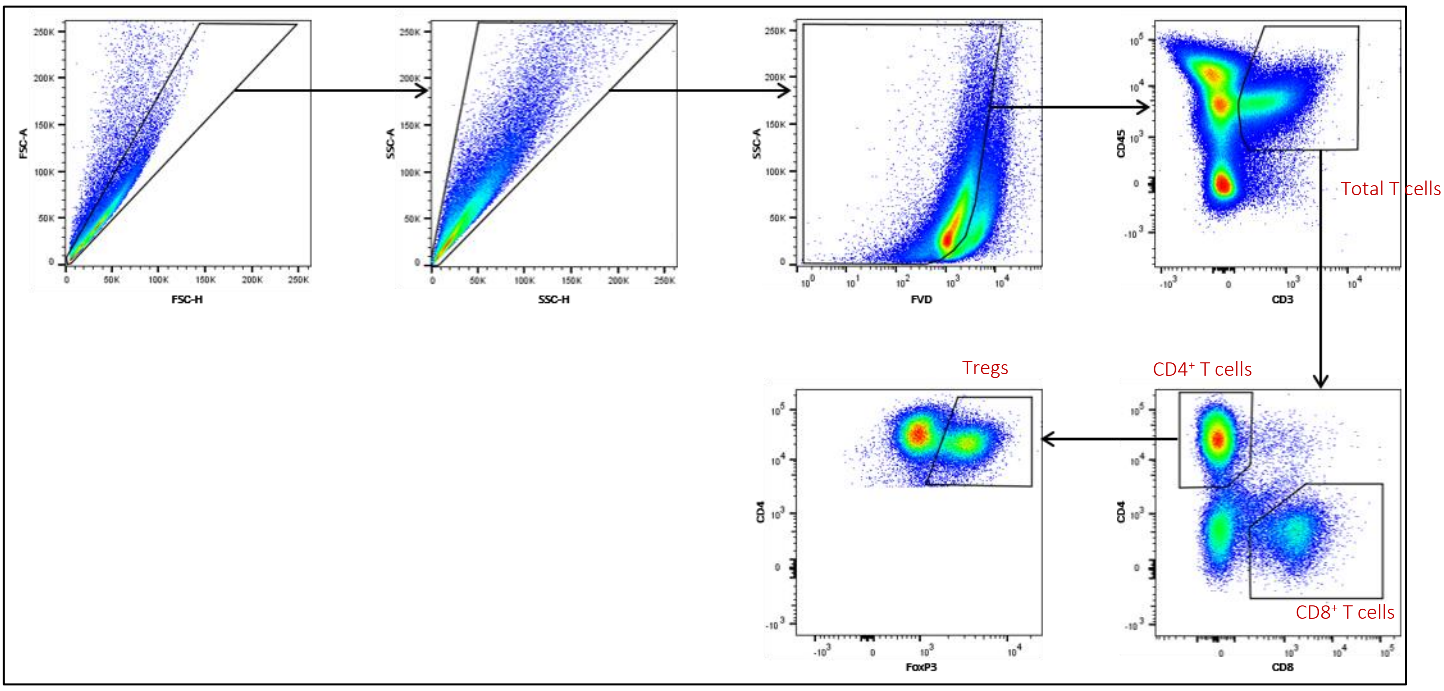

Figure S5d: antibody panels for flow-cytometry

TAMs/MFs and MDSCs

Target	Fluorophore	Clone	Company
CD11b	PerCp Cy5.5	M1/70	eBioscience
CD45	APC	104	eBioscience
Ly6G	Fitc	1A8	BD Biosciences
Ly6C	APC eFluor780	HK1.4	Invitrogen
MHC II	PE-Cy7	M5/114.15.2	eBioscience
F4/80	BV421	T45-2342	BD Biosciences
CD206	PE	C068C2	Biolegend

T cells

Target	Fluorophore	Clone	Company
CD45	APC	104	eBioscience
CD3	APC eFluor780	145-2C11	eBioscience
CD4	PerCP Cy5.5	RM4-5	eBioscience
CD8	BV421	53-6.7	BD Biosciences
FoxP3	AF488	R16715	BD Biosciences

Biology Open • Supplementary information

Nucleus: Area	Cell: Min caliper
Nucleus: Perimeter	Cell: Eccentricity
Nucleus: Circularity	Cell: Channel 1 mean
Nucleus: Max caliper	Cell: Channel 1 std dev
Nucleus: Min caliper	Cell: Channel 1 max
Nucleus: Eccentricity	Cell: Channel 1 min
Nucleus: Channel 1 mean	Cell: Channel 2 mean
Nucleus: Channel 1 sum	Cell: Channel 2 std dev
Nucleus: Channel 1 std dev	Cell: Channel 2 max
Nucleus: Channel 1 max	Cell: Channel 2 min
Nucleus: Channel 1 min	Cell: Channel 3 mean
Nucleus: Channel 1 range	Cell: Channel 3 std dev
Nucleus: Channel 2 mean	Cell: Channel 3 max
Nucleus: Channel 2 sum	Cell: Channel 3 min
Nucleus: Channel 2 std dev	Cytoplasm: Channel 1 mean
Nucleus: Channel 2 max	Cytoplasm: Channel 1 std dev
Nucleus: Channel 2 min	Cytoplasm: Channel 1 max
Nucleus: Channel 2 range	Cytoplasm: Channel 1 min
Nucleus: Channel 3 mean	Cytoplasm: Channel 2 mean
Nucleus: Channel 3 sum	Cytoplasm: Channel 2 std dev
Nucleus: Channel 3 std dev	Cytoplasm: Channel 2 max
Nucleus: Channel 3 max	Cytoplasm: Channel 2 min
Nucleus: Channel 3 min	Cytoplasm: Channel 3 mean
Nucleus: Channel 3 range	Cytoplasm: Channel 3 std dev
Cell: Area	Cytoplasm: Channel 3 max
Cell: Perimeter	Cytoplasm: Channel 3 min
Cell: Circularity	Nucleus/Cell area ratio
Cell: Max caliper	

Table S1. List of 55 parameters used by the software QuPath to detect positive and negative cells.

Biology Open • Supplementary information
